# Gonad Differentiation in the Rabbit: Evidence of Species-Specific Features

**DOI:** 10.1371/journal.pone.0060451

**Published:** 2013-04-08

**Authors:** Nathalie Daniel-Carlier, Erwana Harscoët, Dominique Thépot, Aurélie Auguste, Eric Pailhoux, Geneviève Jolivet

**Affiliations:** UMR 1198, Biologie du Développement et Reproduction, Institut National de la Recherche Agronomique, Jouy en Josas, France; Institut Jacques Monod, France

## Abstract

The rabbit is an attractive species for the study of gonad differentiation because of its 31-day long gestation, the timing of female meiosis around birth and the 15-day delay between gonadal switch and the onset of meiosis in the female. The expression of a series of genes was thus determined by qPCR during foetal life until adulthood, completed by a histological analysis and whenever possible by an immunohistological one. Interesting gene expression profiles were recorded. Firstly, the peak of *SRY* gene expression that is observed in early differentiated XY gonads in numerous mammals was also seen in the rabbit, but this expression was maintained at a high level until the end of puberty. Secondly, a peak of aromatase gene expression was observed at two-thirds of the gestation in XX gonads as in many other species except in the mouse. Thirdly, the expression of *STRA8* and *DMC1* genes (which are known to be specifically expressed in germ cells during meiosis) was enhanced in XX gonads around birth but also slightly and significantly in XY gonads at the same time, even though no meiosis occurs in XY gonad at this stage. This was probably a consequence of the synchronous strong *NANOS2* gene expression in XY gonad. In conclusion, our data highlighted some rabbit-specific findings with respect to the gonad differentiation process.

## Introduction

In mammals, gonad differentiation is initiated early during foetal life. In a first step, genital crests arising from the adjacent mesonephros are colonized by germ cells. Somatic cells and germ cells then differentiate in a coordinated manner: somatic cells differentiate in cells expressing steroids or cells acting as nursing or supporting cells for germ cells. At the same time, germ cells differentiate to acquire the ability to enter meiosis. It is now well established that multiple, complex and reciprocal interactions between all these cell types are mandatory from early foetal life until puberty to ensure the differentiation of fully functional gonads (see [1] for review).

The molecular mechanisms that underlie these events have already been studied intensively in mouse species, because this species is a model of choice for the study of gene function through gene invalidation (for a review see [2]). A cascade of genes has thus been described as the main actors which regulate specific steps. It is now accepted that early events which occur during foetal life profoundly determine the sequence of further gonad development. However, foetal life is extremely short in mice when compared with other mammalian species (especially livestock species such as cows, goats, sheep and pigs) and humans. Even though the same events punctuate overall differentiation, the different milestones are not reached at the same time and probably do not have the same effect in each species. It is therefore difficult to rely on data obtained in the mouse to further explain the origin of aberrations in gonad development that may be encountered in other species, especially in humans.

Rabbit species occupy a particular position among livestock species. Rabbit gestation is longer than that of the mouse (30 days, rather than 21 days in the mouse), but still is short enough to enable regular and systematic sampling throughout foetal life. Meiosis occurs at birth in females and not during foetal life, as is the case in numerous other mammalian species. Moreover, there is a 15-day delay between the appearance of genital crests and the onset of meiosis around birth in the female, while this interval is only 3 days long in the mouse. The rabbit genome sequence is now available and despite some missing annotations, it is easy to extract gene sequences from it. Finally, the recent possibility to modify the genome through specifically targeted nucleases (such as meganucleases, zinc finger nucleases or Transcription Activator-Like Effector nucleases) has provided an opportunity to investigate gene function in the rabbit, as has recently been published by other groups [3].

**Figure 1:**
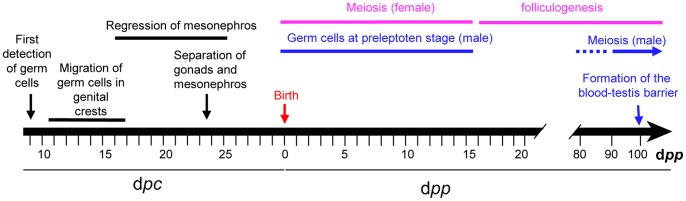
Schematic representation of the principal features of gonad differentiation in the rabbit species.

Numerous histological features have previously been described in detail by other groups; the principal events are summarized in Figure 1. From a macroscopic point of view, rabbit gonads become evident at 14 d*pc*. At this stage, the mesonephros and gonads are still connected and interactions between tissues are probable. A regression of the mesonephros was described from 16 d*pc* to 25 d*pc* [4]. It should be noted that at 23 d*pc*, the gonadal and mesonephric tissues are separated by connective tissue that is supposed to prevent the migration of cells and other substances [5].

In both sexes, the first germ cells are detected from the 9^th^ day *post coïtum* [6]. At 16 d*pc*, most germ cells have already entered the genital crests. In XY and XX gonads, germ cells undergo a series of mitoses, starting from the early stages and lasting until their final differentiation at meiosis [6]–[8]. In XX gonads, the first signs of meiosis have been reported around birth; meiosis spans a period of two weeks after birth. Follicle differentiation initiates after meiosis, as it has been described extensively [9]. In XY gonads, meiotic activity appears by 50 days *post partum* (d*pp*) and becomes abundant by 63 d*pp* [8]. Interestingly, XY germ cells at preleptoten stages have been detected around birth and for two weeks thereafter in the testis, at the same time as meiosis occurs in the ovary, but without any further signs of more advanced meiotic stages [5], [7]. At 10 weeks of age (70 d*pp*), the blood-testis barrier is definitively complete [10]. Puberty is achieved at 5–6 months.

The aim of the present study was to report a first insight into gonad differentiation in the rabbit by studying the expression of genes already characterized as being important actors in sex determination and gonad differentiation in mouse species. A series of genes was retained, as presented in Table 1. At the same time, gonads were analysed histologically throughout the developmental period, alongside immunohistological localizations of proteins when this was possible. Our results showed that the rabbit is a highly interesting species for the study of gonad development during foetal life, as it enables the characterization of original and specific gene expression patterns which coincide better with those seen in human and other livestock species than in mouse species.

**Table 1:** 

	name	primer position	role (mainly in the mouse)
reference genes	H2AFx	no intron	member of histone H2a family
	HPRT	exon 4–5	central role in the generation of purine nucleotides
	YwHaz	exon 5–6	mediates signal transduction by binding to phosphoserine-containing proteins
testisdifferentiation	SRY	no intron	mammalian Y-chromosomal testis-determining gene [13]; already characterized in the chinchilla-rabbit species [14].
	SOX9	exon 1–2	transcription factor, target of SRY and SF1, induces Sertoli cell differentiation, stimulates the expression of AMH [48]; already characterized in the chinchilla-rabbit species [14].
	DMRT1	exon 3–4	transcription factor, interacts with Sox9 [49]; antagonist of Foxl2 [15]; testis differentiation, gametogenesis, expressed by germ cells and Sertoli cells in mice;
	AMH	probably exon 5	Hormone produced by Sertoli cells. Necessary to induce Müllerian ducts regression. AMH gene transcription is activated by SOX9.
ovarydifferentiation	FOXL2	no intron	Transcription factor. FOXL2 loss-of-function in goat is associated with XX sex-reversal [50]. FOXL2, WNT4 and RSPO1 in the mouse are inhibitors of the male pathway [16].
	WNT4	exon 2–3	secreted growth factor, WNT4 and RSPO1 stabilize the β-catenin pathway which contributes to ovary development [51], [52]
	RSPO1	exon 4–5	secreted factor, stimulates the β-catenin pathway, essential to female sex determination [17], [53]
	BMP15	exon 1–2	secreted factor, stimulates folliculogenesis [28]
steroidogenesis	CYP19A1 (aromatase)	exon9–10	enzyme responsible for oestrogen synthesis (irreversible conversion of androgens to oestrogens)
germ cells	OCT4	exon 3–4	transcription factor, pluripotency marker
	VASA (DDX4)	exon 15–16	RNA helicase, critical for germ cell survival and maturation [29] [46]
meiosis	STRA8	exon 6–7	retinoic acid responsive gene; early meiosis marker [32]
	NANOS2	no intron	Zinc finger RNA binding protein, translational regulator, maintains stem cell state during spermatogenesis, Stra8 antagonist [54]
	SPO11	exon 11–12	transesterase that induces double strand breaks during meiosis in mammals (reviewed in [33])
	DMC1	exon 11–12	Recombinase A-related protein (reviewed in [33])

## Materials and Methods

### Sample Collection, Sexing and Staging

New-Zealand (NZ 1077 strain) rabbit embryos and foetuses were collected from the UCEA rabbit facility (Unité Commune d’Expérimentation Animale, Jouy-en-Josas, France). The onset of pregnancy was established as the time of observed copulation. The day of coitus was termed day 0. A minimum of three animals was sampled for each stage from 14 d*pc* until adulthood (6 months). The gonads were rapidly dissected after death, snap frozen in liquid nitrogen and kept at −80°C until RNA extraction. At specific stages (20 d*pc*, 28 d*pc*, 4 d*pp*, 14 d*pp*, 28 d*pp* and in adults), only one gonad was kept for RNA extraction. The other gonad was treated immediately for histological study. Except in 14 and 16 d*pc* samples, the gonads were dissected from the mesonephros. Embryo sexing was performed on the basis of anatomical observation [11] and confirmed by determining the presence of SRY using PCR with head genomic DNA and specific primers (see Table S1).

All experiments were performed with the approval of the local committee for animal experimentation (COMité d’ETHique appliqué à l’expérimentation Animale (COMETHEA), Jouy-en-Josas, accreditation number 12/028). All researchers working directly with the animals possessed an animal experimentation license delivered by the French veterinary services.

### Histology and Immunohistochemistry

Freshly dissected gonads were fixed in 4% paraformaldehyde in phosphate saline buffer (PBS) at 4°C for times ranging between 2 hours and overnight, depending on the size of the tissue. After washing 3 times in PBS, each tissue was transferred successively to 30% ethanol for 1 hour then to 70% ethanol and processed in an automated Shandon Citadel 2000 (ThermoFisher Scientific, Illkirch, France) before being embedded in paraffin wax for histological or immunohistochemical analysis.

For histology studies, all testis or ovary sections were stained with Harris haematoxylin and eosin (H&E) using standard protocols in order to examine basic tissue morphology. For immunohistochemical analysis, 6 µm tissue sections were used. Briefly, tissues sections underwent antigen retrieval by pressure-cooking in 0.01 M Citrate buffer, pH 6.0 for 5 minutes. The sections were then incubated for 2 hours in Blocking Reagent (MOM Kit, Vector Laboratories) and incubated overnight at 4°C with primary antibody diluted in MOM kit buffer (see Table S2 for the list of antibodies and dilutions used). The slides were then washed in PBS and incubated with the appropriate secondary antibody (anti-rabbit IgG Fab2 Alexa Fluor 488 or 555, Cell Signalling; anti-mouse IgG Cy3, Millipore) for 45 minutes at room temperature. The slides were then rinsed in PBS and mounted in Vectashield mounting medium containing DAPI (Vector Laboratories). Observations were performed using a Leica DMRB epifluorescence microscope coupled to a DP50 CCD camera (Olympus).

### Quantitative RT-PCR

Total RNAs were extracted from each gonad using Trizol reagent (Invitrogen Life Technologies, Cergy-Pontoise, France) and the RNeasy mini kit (Qiagen SA, Courtaboeuf, France) according to the manufacturer’s instructions. All samples were treated with DNase I to remove most of the contaminant genomic DNA. Reverse Transcription (RT) was performed on 500 ng-1 µg of total RNA using the High Capacity cDNA Archive kit (Applied Biosystems) and the random primer mix included in the kit.

Quantification was achieved using SYBR Green quantitative PCR (Applied Biosystems) with dilutions of the RT reactions and sets of primers designed by the Primer Express software (Applied Biosystem). Whenever possible, qPCR primers were chosen on separate exons in order to avoid DNA amplification, and all amplicons were 100 base-pairs long. Moreover, for all samples, a RT minus reaction was performed with all RT components except the reverse transcriptase enzyme, and assayed as a complete RT reaction to ensure that no amplification was due to contaminant DNA.

A series of normalizing genes (*β-ACTIN, YHWAZ, HPRT, CPR2, H2AFX*) was tested on all samples for their stable expression in gonad tissues. The GeNorm program included in Biogazelle QBasePlus software (Biogazelle NV, Ghent, Belgium) was used automatically to select a combination of the most stable genes for the rabbit gonad samples. Finally, three genes were used as references for this study (*YHAWZ, H2AFX, CPR2*). In order to correct for inter-run fluctuations, a set of samples was chosen as calibrators and was assayed in all compared runs. Care was taken to consider Ct values within the linear amplification zone. Gene expression was considered as significant when Ct values obtained using 2–5 ng of cDNA in each q-PCR reaction were lower than 34, and when one single DNA fragment with the expected size was amplified as template in each q-PCR reaction.

## Results

### Histological Features Since the Onset of Gonad Development until Early Puberty

**Figure 2:**
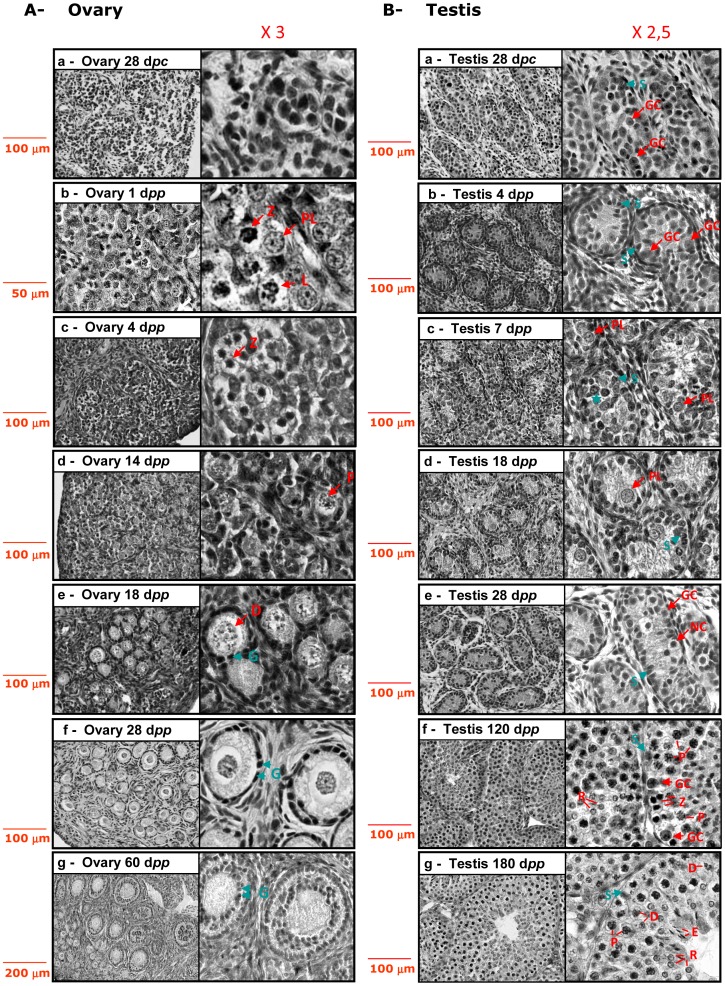
Histological features from the onset of gonad development until early puberty. Paraffin sections of ovaries (2A) and testes (2B) were stained as described in Materials and Methods at 28 d*pc*, 4, 7, 14, 18 and 28 d*pp*, 2 months (ovary, 60 d*pp*), 4 (120 d*pp*) or 6 months *post partum* (testis, 180 d*pp*). Images magnified 2.5× (testes) or 3× (ovaries) are presented for each stage. Arrows point some nuclei harbouring characteristic figures: Sertoli cells (S), granulosa cells (G), germ cells (GC), stages of meiosis (PL = preleptoten; L = leptoten; Z = zygoten; P = pachyten; D = diploten), R = round spermatid, E = elongated spermatid, CN = condensed nuclei. The blue star labels proliferating germ cells (2Bc).

In the present study, sampling for the histological study was initiated at 28 d*pc* in both sexes. At 28 d*pc*, the rabbit ovary was primarily made up of naked oocytes located in nests in the ovarian cortex (Figure 2Aa). After birth, clear figures of meiosis appeared. Nuclei at preleptoten, leptoten and zygoten stages were observed (1 d*pp*, Figure 2Ab). At 4 d*pp*, germ cells were grouped in oocyte nests with zygoten-stage nuclei (Figure 2Ac). As the age of the rabbit increased, more developmentally advanced follicles were detected in the ovary: primordial follicles with squamous granulosa cells at 14 d*pp* (Figure 2Ad), primary follicles with cuboidal granulosa cells at 18 and 28 d*pp* (Figures 2Ae and 2Af), and secondary follicles with several layers of granulosa cells in 2 month-old rabbit ovaries (Figure 2Ag, 60 d*pp*). At the same time, advanced figures of meiosis were observed with nuclei at pachyten and diploten stages.

The foetal testis was clearly distinguishable from the foetal ovary at 28 d*pc*, at which stage seminiferous tubes were individualized (Figure 2Ba). At 4 d*pp*, Sertoli cells and large round germ cells were recognized (Figure 2Bb). We were not able to identify preleptoten stage germ cells in 4 d*pp* testes by opposition to what has been previously described by others [7] [5]. Besides, at 7 and 18 d*pp* (Figures 2Bc and 2Bd), we observed several nuclei of germ cells with preleptoten like figures. At 28 d*pp* (Figure 2Be), we detected no further figures of meiosis; instead, condensed nuclei were observed in few cells. Four (120 d*pp*) and six months after birth (180 d*pp*, Figure 2Bf and 2Bg), differentiated tubes displayed successive layers of spermatogonia, spermatocytes and spermatids.

### Analysis of Gene Expression

As shown in Table 1, a series of genes was studied in order to investigate the molecular mechanisms governing the sex determination and organogenesis of gonads in rabbits. Sampling was initiated at 14 d*pc*. However, at this stage, the genital ridge is still morphologically undifferentiated. Sex determination was thus based on the presence or the absence of the SRY gene, as determined by PCR on genomic DNA.

Moreover, at 14 and 16 d*pc*, the mesonephros and gonads were not dissociated prior to RNA extraction. Consequently, at these stages, the level of expression of gonad-specific genes was probably underestimated.

An attempt was made to localize proteins using immunohistological analysis when a specific antibody was available. However, because most commercially available antibodies are produced in the rabbit species, this method suffered frequently from a high background level; this, combined with the weak sensitivity of this method, meant that it was impossible to confirm the presence of some of the studied proteins at all developmental stages. The immunohistological analysis was thus performed with the aim to localize proteins at the cellular level whenever possible. However, in the absence of histological localization, we have no indication concerning which cells express certain genes.

#### Testis differentiation: SRY, DMRT1, SOX9 and AMH

In mammals, SRY (Sex-determining Region on the Y chromosome) is a major part of a pathway that induces male sex determination [12]. It acts as a transcription factor that regulates the activity of a series of target genes (see for a review [13] and references therein).

**Figure 3:**
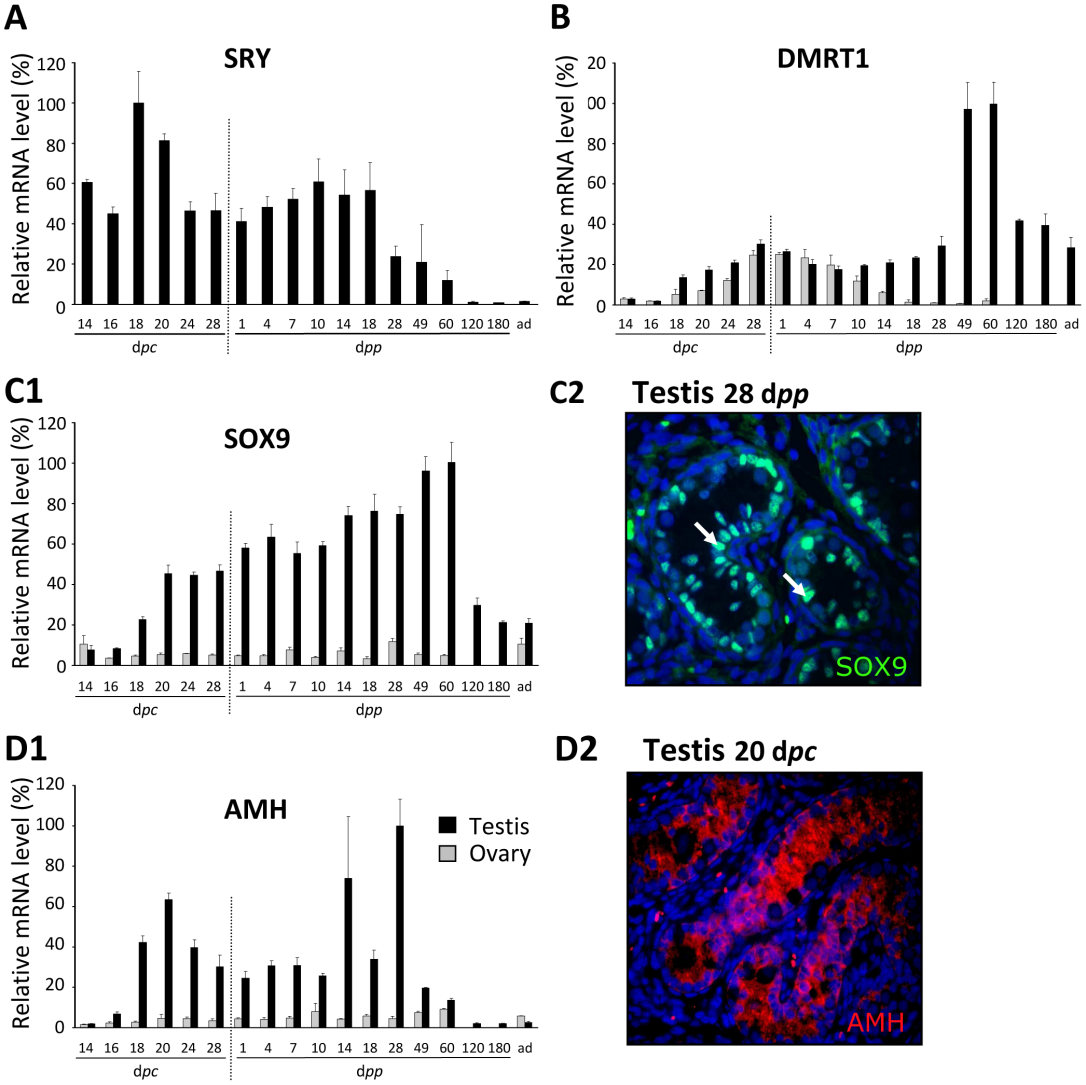
Expression of genes involved in testis differentiation. Expression levels of the *SRY*, *DMRT1*, *SOX9* and *AMH* genes were determined in males (black bars) and females (grey bars) by quantitative RT-PCR, as described in the Materials and Methods. The levels of expression are given as percentages after normalisation to the highest level (100%). Values are means +/− sem of expression levels determined on gonads from at least 3 animals per stage. The horizontal scale indicates the age of the rabbit in d*pc* (days *post coitum*) and d*pp* (days *post partum*). ad = adults, 2-year old rabbit. Note that at 120 and 180 d*pp*, only testes were collected and analysed. **C2, D2**: immunofluorescence detection of SOX9 (C2) and AMH (D2) on paraffin sections of gonads. The antibody against SOX9 specifically stains the nuclei of Sertoli cells (male gonad at 28 d*pp*). The white arrows label some of the Sertoli cells. The antibody against AMH stains the cytoplasm of Sertoli cells (gonads at 20 d*pc*).

In the chinchilla rabbit, *SRY* gene expression was barely but significantly detected by *in situ* hybridization at 13 d*pc* in the genital ridges of XY embryos, and by quantitative PCR on gonad RNA from 14 d*pc* [14]. During the present study using quantitative PCR, significant expression was found throughout foetal life in XY embryos, from the first stage studied (14 d*pc*) to 60 d*pp* (at least 2 months after birth). It further decreased but was still expressed at a significant level in adults (Figure 3A). As anticipated, no significant expression was found in XX gonads.

DMRT1 is a critical regulator of gonad development in mammals, being involved in testis differentiation specifically through preventing female reprogramming in the postnatal testis in the mouse [15]. In the rabbit, *DMRT1* gene expression was detected at significant levels in both XY and XX gonads (Figure 3B). In the latter, expression reached its maximum at the onset of meiosis around birth at 28–30 d*pc,* which was 6 times higher than the level on 16 d*pc*; it then dropped down by 14 d*pp*, to reach an extremely low level in adults. In XY gonads, *DMRT1* gene expression rose from 18 d*pc* until 28–30 d*pc*. A major peak was observed with the first wave of meiosis at around 49–60 d*pp*. The level then remained intermediate until adulthood.

*SOX9* is a mandatory gene for satisfactory development of the male phenotype, and is one of the firstly stimulated genes of the SRY-induced pathway. SRY-induced *SOX9* transcription is a key event in achieving Sertoli cell differentiation. In the chinchilla rabbit, *SOX9* gene expression has been reported in both XY and XX gonads from 13 d*pc* using *in situ* hybridization and from 14 d*pc* using quantitative PCR [14]. In the present study using quantitative PCR, very low levels were found in the ovary compared to the testis (Figure 3C1). The level of *SOX9* gene expression became significantly higher in the testis than in the ovary from 18 d*pc*, rising gradually five-fold between 18 d*pc* and 60 d*pp*, and then falling to reach a basal level similar that seen at 18 d*pc*. As shown by immunofluorescence labelling, the SOX9 protein was only detected in the nucleus of Sertoli cells (Figure 3C2).

The anti Müllerian hormone (AMH) is necessary to induce the regression of Müllerian ducts that otherwise contribute to the female genital tract. The *AMH* gene is one of the targets of the SOX9 gene. In XY rabbit, *AMH* gene expression was low at 14 and 16 d*pc*, had increased by 18 d*pc* (3-4-fold), and peaked at 2–4 weeks after birth (Figure 3D1). Expression of the AMH gene was at its lowest in adults. In XX rabbits, between 14 d*pc* and adulthood, a low level was observed without any significant changes. The AMH protein was detected by immunofluorescence labelling in the cytoplasm of Sertoli cells of 20 d*pc* testis (Figure 3D2).

#### Ovary differentiation: FOXL2, RSPO1, WNT4, CYP19A1

In mammals, a series of genes is expressed specifically in the XX gonad when the male sex determining gene *SRY* is absent. The genes most extensively studied include *FOXL2*, genes of the Wnt/β-catenin pathway (*RSPO1* and *WNT4*), the *CYP19A1* (Aromatase) gene that is required for oestrogen synthesis and the *BMP-15* gene involved in folliculogenesis.

**Figure 4:**
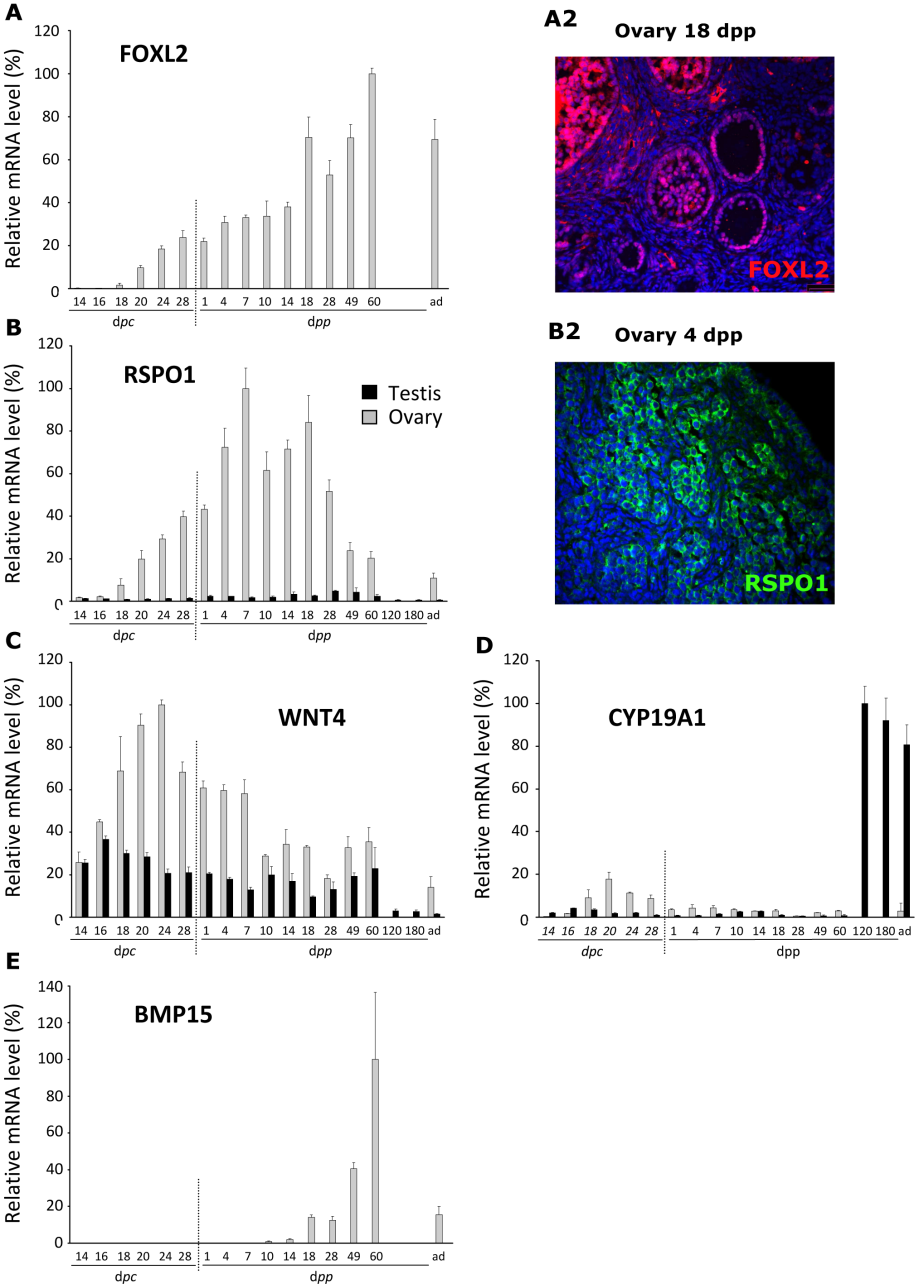
Expression of genes involved in ovary differentiation. Expression levels of the *FOXL2*, *WNT4*, *RSPO1*, *CYP19A1* and *BMP-15* genes were determined in males (black bars) and females (grey bars). Values are given as percentages after normalisation to the highest value (100%). The horizontal scale represents the age of animals in d*pc* and d*pp*. ad = adults, 2-year old rabbit. Note that at 120 and 180 d*pp*, only male samples were collected. **A2, B2**: immunofluorescence detection of FOXL2 (A2) and RSPO1 (B2) on paraffin sections of gonads. The antibody against FOXL2 specifically stains the nuclei of granulosa cells (female gonad at 18 d*pp*). The antibody against RSPO1 specifically stains the cytoplasm of germ cells (female gonad at 4 d*pp*).

*FOXL2* is a mandatory gene for female sex determination that maintains the ovarian phenotype through an active process [16]. In the rabbit, the testis did not significantly express the *FOXL2* gene. In females, the first signs of significant *FOXL2* gene expression were detected at around 16–18 d*pc* (Figure 4A). By 20 d*pc* until adulthood, the level of *FOXL2* gene expression rose gradually to reach a maximum that was sustained in adults. FOXL2 was clearly detected by immunohistofluorescence analysis in granulosa cells (Figure 4A2, 14 d*pp*).

In the mouse, WNT4 and R-spondin-1 (RSPO1) promote ovarian fate and block testis development through induction of the β−catenin pathway [17]–[19]. In the rabbit, the two genes are expressed in the gonads of both sexes from the early stages of differentiation (14–16 d*pc*; Figures 4B and C). Interestingly, from 18 d*pc*, the expression levels of both genes became higher in ovary than in testis. In females, *WNT4* gene expression reached its maximum at 24 d*pc* and then fell gradually to reach a low level in adults. *RSPO1* gene expression peaked later than *WNT4* gene expression, at 7 d*pp,* one week after birth, then decreased to a minimal level in adult gonads. RSPO1 was detected by immunofluorescence in the cytoplasm of germ cells in the cortical zone at 4 d*pp* (Figure 4B2).

The cytochrome P450 aromatase is responsible for the irreversible conversion of androgens into estrogens. In goat and fish, the *CYP19A1* gene encoding the aromatase is a target of the FOXL2 transcription factor that acts on regulatory elements located on the *CYP19A1* gene promoter [20]–[22]. In the rabbit, aromatase activity has been characterized by other groups *in vitro* by measuring the ability of the embryos to convert androgens into oestrogens. Thus two windows of aromatase activity have already been observed: the first in pre-implantation embryos (male and female) at 6–11 d*pc* [23]–[26] and the second in XX but not XY gonads at 20 d*pc* [27]. In the present study, *CYP19A1* gene expression was extremely low in testis during foetal and perinatal life (Figure 4D). By contrast, in ovary, it peaked at 20 d*pc* and then returned gradually to lower values by 28 d*pp*. Moderate *CYP19A1* gene expression levels were observed in adult XX rabbits. Interestingly, very high levels of aromatase gene expression were detected in the testis 4 months after birth (120 d*pp*) and in older animals (180 d*pp* and older adults).

The BMP-15 factor is an ovary-specific secreted factor that acts in a paracrine manner. It is known to play a fundamental role in the growth and maturation of follicles from primordial to primary, and subsequent secondary follicles [28]. In the female rabbit, the growth of primordial follicles starts about two weeks after birth, at the end of meiosis. Significant *BMP-15* gene expression was found in ovaries from 14 d*pp*, when primordial follicles were clearly detected (Figure 4E). The highest level was measured at 60 d*pp*. A lower level was detected in adults. As expected, the *BMP-15* gene was not expressed significantly in testes.

#### Expression of germ cell marker genes

**Figure 5:**
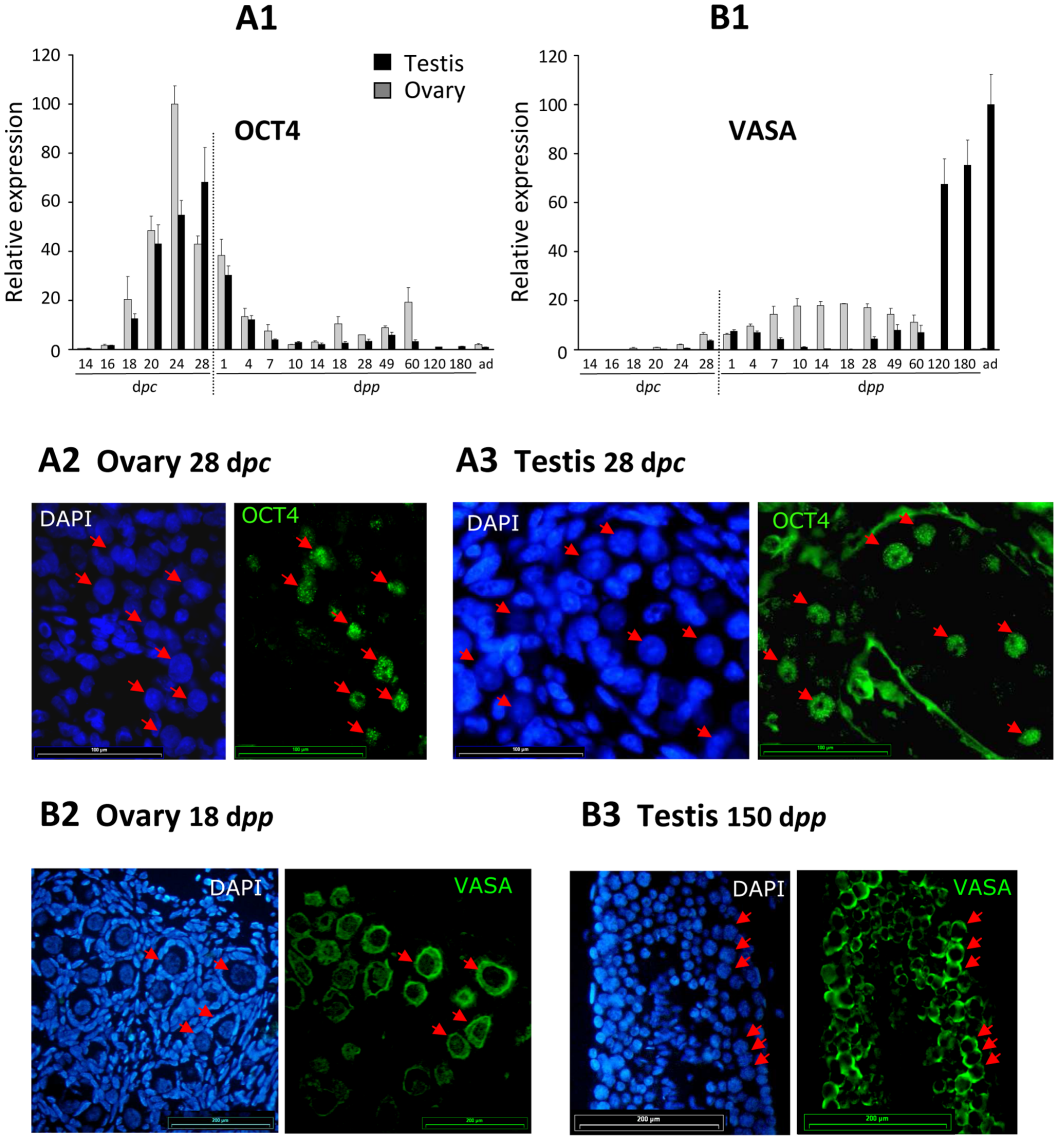
Expression of germinal-specific marker genes. Expression levels of the *OCT4* and *VASA* genes were determined in testes and ovaries. Values are given as percentages after normalisation to the highest value (100%). A2, A3 and B2, B3: immunofluorescence detection of OCT4 and VASA proteins in paraffin sections of gonads. Immunofluorescence pictures are given with each DAPI counterstaining. The antibody against OCT4 stains the nuclei of germ cells in 28 d*pc* testis (A2) or ovary (A3). Using the antibody against VASA, a cytoplasmic fluorescence is visible in germ cells in 18 d*pp* ovaries (B2) and 150 d*pp* testes. Red arrows point to the specifically labelled nuclei. Horizontal bars represent 100 µm (A2 and A3) or 200 µm (B2 and B3).

OCT4 is commonly used to delineate the germ stem cell population, since it is characteristic of totipotency. In the rabbit, we observed that the *OCT4* gene was significantly expressed at all studied stages and in both sexes (Figure 5A1). The level of expression rose from 14 d*pc* in the early-differentiated gonad in line with the number of germ cells. In both testes and ovaries, the maximum level of expression was found at around 24–28 d*pc*, while the number of germ cells was the highest at 26 d*pc* [6]. From 28 d*pc* in ovaries and 1 d*pp* in testes, *OCT4* gene expression declined gradually to stabilize in adults at a level similar to that seen at the early stages. As expected, the OCT4 protein was localized in the nuclei of germ cells (Figure 5 A2 and A3, in ovaries and testes, respectively). Interestingly, despite a significant expression of the *OCT4* gene in young rabbits and adults, we never succeeded in immuno-labelling any positive cells after birth in either testes or ovaries. This was probably due to the fact that pluripotent germ cells which still express the *OCT4* gene after birth are too rare to be visualized.

Previous studies in the mouse had characterized a number of genes whose expression is necessary for germ cell survival and maturation in both sexes [29]–[31]. One such gene is the *Mvh* gene (mouse VASA homolog). In the rabbit, *VASA* gene expression was observed in both sexes in all the samples studied. The lowest expression was found at 14 and 16 d*pc* (Figure 5 B1). It reached a first peak after birth, at around 4 d*pp* in the testis and 14 d*pp* in the ovary. In the testis, the level of expression then fell at around 10–18 d*pp*, rising subsequently to being the highest in the adult gonad. The VASA protein was detected by immunohistology in germ cells only with a cytoplasmic location (ovary 18 d*pp* Figure 5B2 and testis 150 d*pp*, Figure 5B3).

#### Mechanisms of meiosis induction and the retinoic acid transduction pathway

In the female rabbit, the onset of meiosis occurs around birth. The first signs of leptoten stages are visible at 26–28 d*pc*, and later stages are clearly observed from birth. Meiosis spans for two weeks after birth. Finally, ovocytes are blocked at the diploten stage [5]. In XY rabbit, several authors have reported figures of germ cells in the preleptoten stage before birth, at the same stage as in the female, without any signs of further true meiosis; however, the first signs of true meiosis were only observed in the male 2–3 months after birth [5], [7], [8].

**Figure 6:**
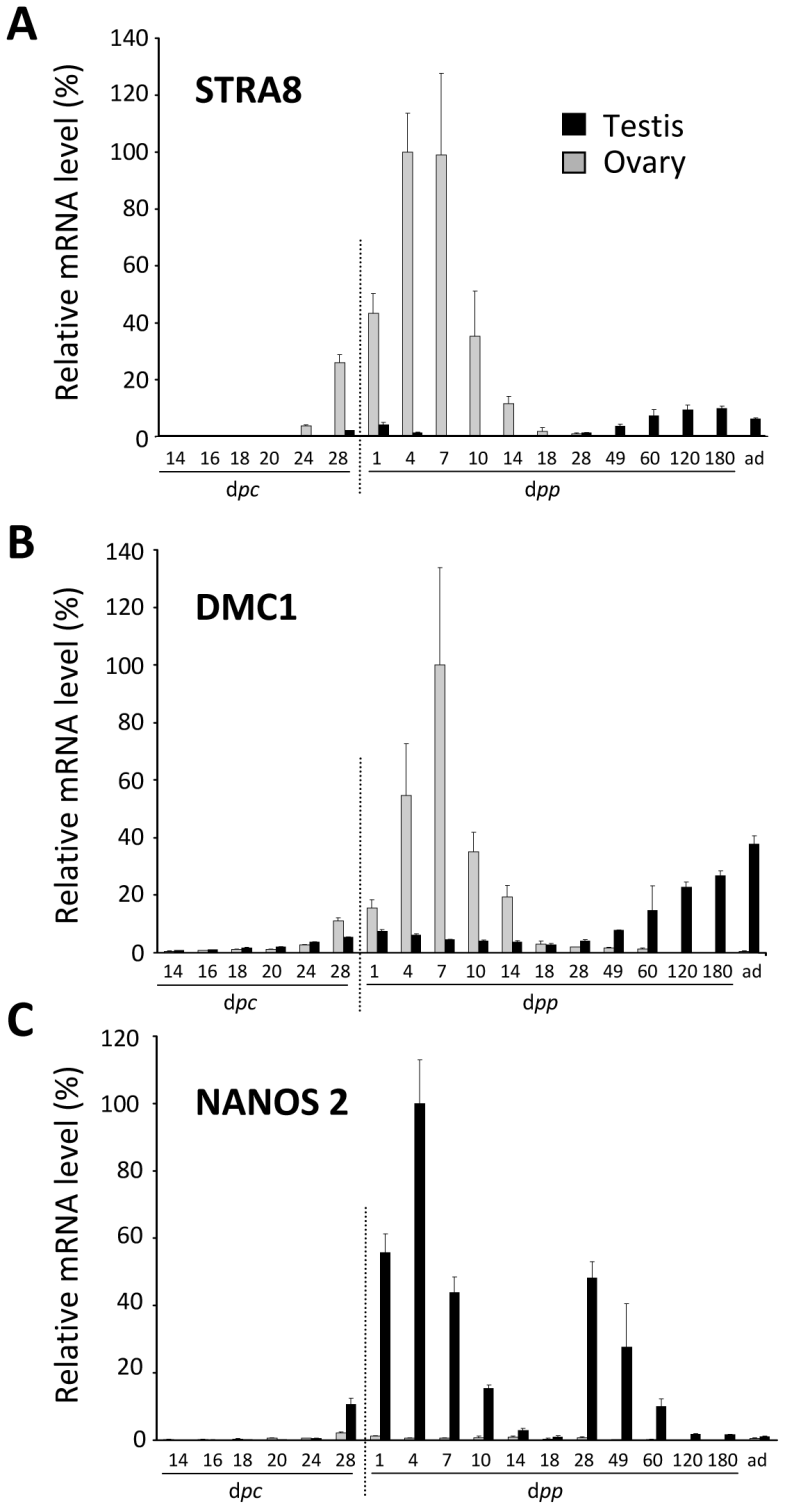
Expression of genes involved in meiosis. Expression levels of the *STRA8*, *DMC1* and *NANOS2* genes were determined in male and female gonads. Values are given as percentages after normalisation to the highest value (100%). Note that at 120 and 180 d*pp*, only male samples were collected.

In this study, the expression of genes specific to meiotic events was analysed throughout gestation until adulthood. Three genes were studied in particular (Figure 6). The *STRA8* gene is expressed under retinoic acid (RA) activation, and is known to be mandatory for the onset of meiosis [32]. DMC1 is a recombinase A related protein necessary for the progression of meiosis [33]. In the mouse, NANOS2 is essential for the survival of primordial germ cells, and it has been suggested that it acts in the testis as a meiotic repressor [34].

As expected, the *STRA8* gene was significantly expressed in the ovary from 24–28 d*pc*, at the onset of meiosis (Figure 6A). Its expression was maintained throughout meiosis, and then fell after the arrest of meiosis on day 14 *pp*, after which it was no longer significantly expressed. The pattern of expression of the *DMC1* gene was roughly similar to that of the *STRA8* gene. A significant but extremely low level of *NANOS2* gene expression was observed in the ovary from 24 to 14 d*pp*. At other stages, the *NANOS2* gene was not significantly expressed in the ovaries.

In testes, the *STRA8* and *DMC1* genes were clearly expressed when meiosis started, from 49 d*pp* until adulthood. Surprisingly, a low but significant expression of the *STRA8* and *DMC1* genes was observed from 24–28 d*pc*, and was maintained for approximately 14 days after birth. Thus, at the time of onset of meiosis in the ovary, genes specific to the induction and advancement of meiosis were also expressed significantly in the testis. Interestingly, a peak of *NANOS2* gene expression was observed from day 28 d*pc*, immediately after the peak of *STRA8* gene expression. This peak was followed by a decrease (day 10–14 d*pp*), and then a second peak (day 28 d*pp*).

## Discussion

The present study reports on a first transcriptomic analysis of gonads in the rabbit throughout development, from 14 days post-conception to adulthood. Numerous studies have been performed to date in the mouse to investigate the gene pathways involved in sex determination and gonad differentiation mechanisms, and to achieve a temporal profiling in somatic and germ cells RNAs such as that reported recently ([35] and references therein). But despite the huge quantity of data that has accumulated in recent years, backed by functional genomic studies in the mouse, several authors have suggested that the mouse model is not wholly relevant to other mammalian species. As reported by others ([36] [14] and references therein), a Carnegie stage comparison of the major developmental stages in male sexual development across several mammalian species showed that genital ridge emergence, *SRY* and *SOX9* gene expression, and testis cord formation occur at an earlier stage in the mouse than in all other species. Furthermore, in the other mammals studied, such as the rabbit species, these events were reported at comparable developmental stages. Considering the length of gestation, the occurrence of meiosis at around birth in the female, the high number of foetuses in the litter and recent opportunities to investigate gene function in non-mouse species by using enzymes designed to target specific regions of any genome, the rabbit species could be used conveniently to study the gene pathways involved in sex determination and gonad development.

**Figure 7:**
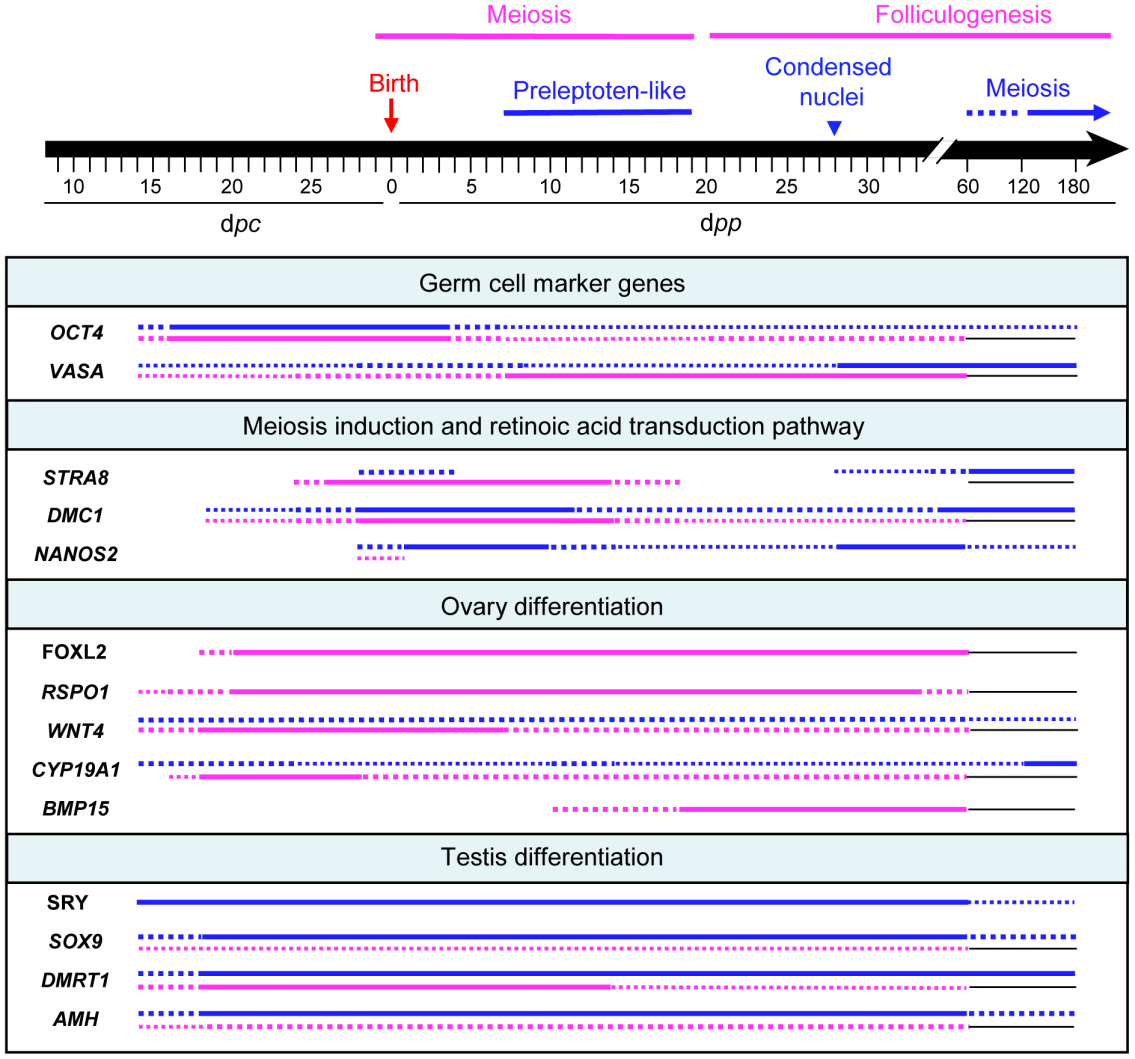
Schematic representation of gene expression patterns and main histological observations. Continuous lines represent a significant gene expression in ovaries (pink) or testes (blue) extracts. Dotted lines indicate that expression is significant but at a lower level. Thin black lines are drawn when no samples were assayed.

The main features of the present study have been reported in a schematic conclusion figure (Figure 7). Based on our present data, we are able to confirm in the rabbit the relevance of the classic model for mammalian sex determination. Indeed, as it is clearly visible in Figure 7, in the testis, expression of the *SRY* gene started from 14 d*pc* just after genital crest individualization, together with that of the *DMRT1, SOX9* and *AMH* genes, while in the ovary, the onset of expression of the *FOXL2, RSPO1* and *WNT4* genes marked the female commitment of the gonad from 18 d*pc*.

However, it is worth highlighting some specific features of the rabbit, as reported in the present study. The patterns of *SRY* and *SOX9* gene expression have already been compared by others in the mouse [37], chinchilla-rabbit [14], goat [38] and cow [36]. The authors distinguished two kinds of profiles: the mouse profile, with a short peak of *SRY* gene expression during early fetal life and no expression later; and the other profiles with a low but sustained *SRY* expression shortly after the early fetal peak until adulthood. Our present study indicates that the rabbit *SRY* gene expression profile is closer to the chinchilla-rabbit, goat and cow profile. However, in the rabbit, the *SRY* gene expression is remarkably high for two months after birth, which is not the case in the other species. To date, the physiological importance of this high level is unknown.

The pattern of aromatase gene expression also necessitates some comments. i) In the ovary, our study highlighted a peak of aromatase gene expression at around 18–20 d*pc*, followed by a gradual decline. Such a peak of aromatase gene expression has previously been reported in the goat at the onset of ovary differentiation [20]. Likewise, a peak of aromatase activity has been described at this stage in the rabbit [27], sheep [39] and humans [40], but not in the rat or mouse [41]. To date, the physiological importance of this early aromatase activity – or of the peak of aromatase gene expression – remains unknown. ii) Unlike what has been described in the goat [20], [42], our study did not report any increase in aromatase gene expression during follicular growth. In the rabbit ovary, we reported a lower level after meiosis than during foetal life, while in the goat, the principal peak of aromatase gene expression was observed during the growth of follicles, and was 4–5 times higher than that observed during foetal life. However, it should be pointed out that in the rabbit, the level of aromatase gene expression was far from being null throughout follicle growth and differentiation. Consequently, it can be assumed that aromatase probably plays an important role after meiosis in the gonad development of young rabbits, as has already been proposed in other species. iii) The level of aromatase gene expression was extremely high in the rabbit testis late in puberty (120 d*pp*) and in the adult. Unfortunately, we were not able to localize the protein by immunohistochemistry using commercially available antibodies. Since the level of circulating oestrogens is very low in the male rabbit, it is reasonable to suppose that aromatase gene-expressing cells are enclosed within the seminiferous tubules. Thus the presence of the blood-testis barrier that appears at 70 d*pp* in the rabbit [10] could explain why the oestrogens produced are not secreted in peripheral blood. Additional experiments need to be performed to determine the role of oestrogens produced by the testis in the adult rabbit, but this could be involved in the later process of spermatogenesis, as has already been described in several species (see a review in [43]). To conclude on aromatase data, it is of importance to undertake a further study in order to elucidate the role of this enzyme in rabbit gonad differentiation during fetal and adult life. Experiments are in progress in our laboratory through aromatase gene invalidation in this species.

Expression of the *BMP-15* gene was studied in order to investigate a gene that has been shown by others to be involved in follicle growth in many other mammalian species [44], [45]. Here, we report a *BMP-15* mRNA profile in complete agreement with this hypothesis, since *BMP-15* mRNA was detected in the ovaries from 14 d*pp* when primordial follicles were observed, peaked during follicle growth and diminished thereafter.

As expected from previous studies in the mouse, and as we observed by immunofluorescence, *OCT4* and *VASA* are germ cell-specific genes. *OCT4* gene expression varied with the number of undifferentiated germ cells. In ovaries, it was high until 28 d*pc*, and dropped down around birth, when in the mouse *OCT4* gene expression is down regulated long before birth. This apparent discrepancy is due to the fact that the rabbit is a species presenting a delayed XX meiosis, compared to the mouse, with the first signs of meiosis at 28 d*pc* in the ovaries, just before birth and not as in the mouse during fetal life. It is thus not surprising that the *OCT4* gene expression decreased around birth, which coincides with germ cell differentiation at the onset of meiosis. Interestingly, in testes also, the expression of *OCT4* fell dramatically around birth. However, meiosis starts two-three months after birth in testes. Thus it can be hypothesised that XY germ cells were committed to a differentiation pathway at around birth and onwards, even in the absence of meiosis.

*VASA* gene expression, which is characteristic of germ cells in the mouse from early stages [46], was detected by RT-PCR in the earliest studied stages in the rabbit. It increased gradually as *OCT4* gene expression declined, in both testes and ovaries. As already shown in the mouse, in the rabbit also this probably reflected the number of germ cells under differentiation. In ovaries, its expression fell after meiosis. As found by other authors, this corresponds to a phase of germ cell degeneration around meiosis. In rabbit testes, a phase of germ cells degeneration has been described a few days after birth [7]. This phenomenon was probably responsible for the decrease in *VASA* gene expression in the testis that we reported around birth. The subsequent rise in *VASA* gene expression from 28 d*pp* until adulthood in the testes probably underlines the progress of mitosis in numerous germ cells, which precedes meiosis at the end of puberty.

Of the various genes that characterize meiosis, we chose to study three genes: *STRA8*, which is characteristic of the RA-dependent induction of meiosis; *DMC1*, which encodes a protein involved in the mechanism of meiosis; and *NANOS2*, which is supposed to be involved in the repression of meiosis in the testis. In the rabbit ovary, *STRA8* and *DMC1* genes were expressed significantly around birth and for two weeks after, as meiosis progressed, but not after that. The model for the RA induction of meiosis is thus probably acceptable in the rabbit ovary, as has previously been demonstrated in the mouse.

Interestingly, we observed a low but significant expression of *STRA8* and *DMC1* genes around birth and during a few days after birth in rabbit testes. Considering that RA induces meiosis in both sexes, this probably indicates that in the testis, an RA-dependent stimulus could induce meiosis in the same way as in ovaries around birth. This concurs with previously reported observations of XY germ cells at the preleptoten stage in rabbit testes at around birth and for two weeks after [5], [7], [8]. In the present study as well, the presence of preleptoten like figures at 7 and 18 dpp indicates that meiosis was initiated in the testes but aborted thereafter since no more advanced stages of meiosis were further detected. Consequently, it should be of importance to study genes involved in the synthesis or the metabolism of RA in the rabbit gonad to further clear up the role of the RA induced pathway, as it has been elegantly done in the mouse [47].

In the mouse, it has been supposed that the *NANOS2* gene is responsible for the absence of meiosis in the foetal testis [34]. In the rabbit testis also, the *NANOS2* gene may be at the origin of meiosis arrest. Interestingly, the pattern of *NANOS2* gene expression displayed two peaks, like the pattern of *VASA* gene expression. This was not surprising because it has been described in the mouse that both the *NANOS2* and *VASA* genes are expressed by germ cells. This means that the *NANOS2* gene was expressed in rabbit XY germ cells in a sustained manner from few days before birth (24–28 d*pc*) until the first signs of meiosis at the end of the pre-puberty period (60 d*pp*). Finally, it is important to point out that in the rabbit ovary, the *NANOS2* gene was expressed significantly as in the testis from few days around birth and for two weeks afterwards, but at an extremely low level. This could suggest that in the ovary, *NANOS2* gene expression is repressed around birth and subsequently, while this is not the case in the testis.

In conclusion, this study has provided some detailed findings regarding gene expression by the rabbit gonads during early foetal life until adulthood, in both sexes. Most of the genes that have already been characterized in other species (essentially the mouse) as mandatory actors in gonad differentiation were studied, showing that in the rabbit also, they probably play fundamental roles. Notably, the length of the foetal period between the formation of genital crests and birth is sufficiently long (15 days) to offer an opportunity to investigate this period in detail. However, further studies now need to be performed in order to take advantage of this uniqueness of the rabbit species and gain insight into some of the gene pathways involved in the mechanisms of gonad differentiation, such as the aromatase gene pathway in the foetal female gonad or the RA-induced pathway involved in the induction and progress of meiosis.

## Supporting Information

Table S1 (XLS)

Table S2 (XLS)
